# Relational counselling as a psychosocial intervention for dementia: Qualitative evidence from people living with dementia and family members

**DOI:** 10.1177/1471301220984912

**Published:** 2020-12-31

**Authors:** Alys Wyn Griffiths, Emily Shoesmith, Cara Sass, Paul Nicholson, Divine Charura

**Affiliations:** School of Health & Community Studies, Leeds Beckett University, Leeds, UK; School of Health & Community Studies, Leeds Beckett University, Leeds, UK; Department of Health Sciences, University of York, UK; School of Health & Community Studies, Leeds Beckett University, Leeds, UK; Leeds Institute of Health Sciences, University of Leeds, UK; School of Health & Community Studies, Leeds Beckett University, Leeds, UK; School of Health & Community Studies, Leeds Beckett University, Leeds, UK; School of Education, Language & Psychology, York St John University, UK

**Keywords:** psychotherapy, therapeutic relationship, cognitive impairment, psychosocial interventions, relational counselling

## Abstract

Counselling and other psychotherapeutic interventions can be offered to people with dementia and their caregivers, to treat specific conditions or symptoms (e.g. affective disorders such as depression). Psychotherapeutic interventions also offer the opportunity for individuals with dementia and their families/caregivers to engage in psychological support for a wide range of presentations. However, little is known about how those within this demographic who receive these interventions perceive the experience. This study aimed to understand the experiences of individuals with dementia or caring for someone with dementia, before and after a 12-week relational counselling intervention delivered through a third sector organisation within England. Semi-structured interviews were completed with participants (29 pre-intervention and 25 post-intervention). Framework analysis was conducted, with four main themes identified; expectations and outcomes of counselling, emotional impact of life with dementia, appraisals of identity and importance of therapeutic relationship. Participants reported that counselling interventions addressed a range of needs and concerns that they had, enabling them to reassess and reconsider these. Specific training is needed before therapists deliver therapeutic interventions with people with dementia, to ensure that appropriate support is provided for those with more severe cognitive impairment or who may have fluctuating capacity. Future research should explore the experiences of people with dementia and their caregivers, across different counselling modalities, to establish the appropriateness and effectiveness of relational counselling.

## Background

The need to provide accessible post-diagnostic services and support for people with dementia and their families/caregivers has been widely recognised (e.g. UK, Department of Health, 2015; Canada, Public Health Agency of Canada, 2019). Whilst there is no cure for dementia, providing non-pharmacological and individualised support is imperative (Aminzadeh et al., 2007). Psychosocial treatments have been poorly understood and implemented, although the demonstrated value of these interventions continues to grow (Oyebode & Parveen, 2019). Counselling interventions, which involve regular dialogue and therapeutic encounter between an individual and a counsellor/therapist, usually for a set number of sessions, can impact how individuals respond to their diagnosis and experience of dementia (Bryden, 2002).

Both people living with dementia and their caregivers are thought to be particularly susceptible to experiencing anxiety and/or depression (Enache et al., 2011; Garre-Olmo et al., 2016). Learning to live with a diagnosis of dementia is a complex process, and willingness to express emotions associated with this process often poses a challenge for some people with dementia (Weaks et al., 2015). Additionally, anxiety and/or depression is thought to arise due to the person experiencing cognitive decline and the emotional regulation difficulties often associated with this (Kiosses et al., 2015). Therefore, delivering psychotherapeutic interventions that address one’s lived experience, for example working through feelings of loss, isolation, role change, the challenge of symptoms and being understood within this process could improve the quality of life of those living with dementia (Scholl & Sabat, 2008). Previous research has highlighted the need for emotional and practical support post-diagnosis (Kelly & Innes, 2016) and the importance of post-diagnostic counselling for people with dementia to facilitate working through the impact of and acceptance of their diagnosis (Birtwell & Dubrow-Marshall, 2018). A recent systematic review (Shoesmith et al., 2020) found that psychotherapeutic interventions can lead to meaningful change for both people with dementia and their caregivers, although highlighted that considerably more evidence has considered caregivers.

Conversely, counselling for caregivers is well established and can often offer direct relief through conversation (Grossfeld-Schmitz et al., 2010). Previous research has reported that counselling may prevent a decline in health in caregivers of people with dementia (Mittelman et al., 2007), reduce anxiety/depression (Hamill & Mahony, 2011; Jimenez & Gray, 2006; Losada et al., 2015) and enhance their coping skills with the caregiving role (Behrndt et al., 2019).

Despite this, very little is known about the experiences of attending counselling for those living with, and those supporting those with, dementia. Much of the research conducted which offered psychotherapeutic interventions has focussed on quantitative data. To date, mixed evidence has been found for the benefits of counselling for people with dementia and their caregivers. Most interventions have adopted either a problem-solving or Cognitive Behavioural Therapy approach and have demonstrated benefits for outcomes including depression, apathy and coping. However, several other studies have demonstrated a lack of efficacy for counselling on any outcomes (see (Shoesmith et al., 2020) and Tay et al., 2019 for review). Despite these inconsistencies, there remains a paucity of literature about the perspective of the participants, their lived experiences and expectations of counselling and perceived barriers and facilitators to engaging with therapeutic interventions (Elvish et al., 2014).

Previous research has argued that identifying important aspects of psychotherapeutic interventions from participant perspectives will improve treatment and provide understanding of barriers and facilitators to engagement in the counselling process (Birtwell & Dubrow-Marshall, 2018; Berg et al., 2008). To deliver effective counselling to people with dementia and their caregivers, it is vital to consider the individuals’ lived experiences and perspectives, and by doing this, services can subsequently be tailored accordingly (Elvish et al., 2014). One model highlighted nine therapeutic tasks that therapists should conduct with people with dementia (Weaks et al., 2009). This includes exploring what ‘normal life’ is, understanding changes in roles and relationships within support networks, challenging and embracing stigma around dementia diagnosis and navigating the health system (Weaks et al., 2009). This provides a comprehensive framework for conducting work in this area and provides a basis for understanding the challenges faced by people living with dementia.

This study specifically considered a relational approach to counselling, which highlights the importance of the therapeutic process and relationship, allowing a range of techniques and therapeutic approaches to be integrated by the therapist (Erskine, 2015; Finlay, 2016). Relational approaches focus on the emergent, here-and-now relationship between therapist and client, where the therapist flexibly works with each individual’s needs (Erskine, 2015; Finlay, 2016), with the most important component being the relationship between therapist and client. The approach considers the complex and diverse needs of individuals (Paul & Charura, 2014), therefore being inclusive of people with dementia. Therefore, the aim of the present study was to understand the experiences of individuals with dementia or caring for someone with dementia, before and after a 12-week relational counselling intervention.

## Methods

### Procedure

The counselling intervention ran for 18 months within a faith-based community organisation, staffed by one counsellor offering a course of 12 weekly counselling sessions lasting 1 hour. Counselling recipients were referred to the service through their family doctor or third sector agencies. Sessions were free to attend, and in some cases, the overarching organisation funded transport to appointments. The service was provided as part of a funded project to establish a range of supportive provisions for people affected by dementia in the local community and ran alongside a weekly programme of activity programmes and peer-led support for family caregivers. Individuals attending the counselling service were recruited. Initial contact was made by someone working within the service who provided the research team with contact details of those willing to be contacted about the study. Only individuals with dementia who had capacity to provide consent (as judged by the service) were approached. All participants provided written informed consent prior to participation, and ethical approval was obtained from the Leeds Beckett University Ethics Committee.

Semi-structured interviews were conducted immediately before the first counselling session and within 2 days of the final counselling session. A topic guide was developed by the research team and used at each time point, with the first guide containing questions relating to the participants’ background, reasons for accessing support and expectations of counselling. Post-counselling, participants were asked whether the sessions had met their expectations and identified any changes in their lives as a result of accessing support. Researchers also framed questions to each participant’s experiences by re-reading their transcript from the first interview and utilising this information where appropriate. Interviews were conducted at the third sector service, at participants’ homes or over the telephone, dependent on participant preference with one of the research team (CS or ES). The first interview focussed on reasons for seeking counselling, expectations for counselling and current experiences. The second interview reflected back on these issues to examine whether expectations had been met and explored participants’ counselling experiences. Interview length ranged from 8 minutes to 58 minutes, with a mean duration of 31 minutes. Interviews were audio recorded and transcribed by CS.

### Participants

Inclusion criteria included the following: (1) a diagnosis of dementia or supporting someone with a diagnosis of dementia, (2) capacity to provide consent (as judged by the service) and (3) ability to communicate verbally in English. A total of 29 individuals participated in an interview before they began counselling, and 25 also participated after their final session. Six participants were living with dementia, and 23 supported someone with dementia. This included three ‘dyads’, whereby both the person with dementia and their respective caregiver attended sessions. The sample imbalance was due to several factors, including that one of the main referral pathways was a local carer support network, relatives seemed more likely to seek counselling than the individual with dementia themselves and people with dementia were not always aware of the service.

Participants were mainly female (*n* = 22, 76%) and identified as white British (*n* = 26, 90%). For participants with dementia, the average age was 81 years, and the majority had a diagnosis of Alzheimer’s disease (*n* = 4, 61%). Of those who completed the Montreal Cognitive Assessment cognitive screening measure (Nasreddine et al., 2005) pre-intervention, (*n* = 3) two scored 21 indicating mild dementia and one participant scored 16 indicating the presence of more severe cognitive impairment. For caregivers of people with dementia, 11 participants (48%) supported their spouse, and 12 participants supported their parent (52%) (see Table 1 for full demographics).

**Table 1.** Participant demographics.

People with dementia (*n* = 6)		
Gender	Female	3 (50%)
Mean age (years)		81 (range = 61–95)
Ethnicity	White British	4 (68%)
	White European	2 (32%)
Diagnosis	Alzheimer’s disease	4 (64%)
	Vascular dementia	1 (16%)
	Mixed dementia	1 (16%)
Montreal Cognitive Assessment (n=3)	Mean	19.33
	Range	16–21

Caregivers (n = 23)		
Gender	Female	19 (83%)
Ethnicity	White British	22 (95%)
	Black British	1 (5%)
Relationship with person	Spouse	11 (48%)
	Parent/child	12 (52%)

### Intervention

A 12-week intervention of weekly one-hour relational counselling sessions was delivered. The intervention aimed for the therapist to be emotionally present offer a professional and supportive relationship through listening and working through whatever the person with dementia or those who care for someone with dementia wanted to explore. The therapist had completed DipHE Therapeutic Counselling and stated that they had not received any formal training in supporting people with dementia; instead, this had been developed through personal and professional experience. Participants were asked to visit the third sector organisation, but for those who were unable to, the therapist visited them in their own home. No dyadic sessions were delivered, even where both individuals were receiving the intervention at the same time. Dyadic sessions involve the person with dementia and their caregiver attending joint sessions, usually with the overarching aim to strengthen the relationship (Whitlatch et al., 2006).

### Data analysis

Qualitative data were analysed using NVivo 12. Framework analysis (Smith & Firth, 2011) was used to identify and develop core themes. The research team developed an initial coding framework which guided and created a structure for further data analysis. Each transcript was independently coded and analysed by one author. Subsequently, all of the authors discussed their analysis and reached agreement on where quotes should be placed within the framework.

## Findings

For two participants, the intervention was delivered across 14 weeks due to holidays and illness. Four main themes were identified, with a number of sub-themes (see Table 2). Quotations are presented to illustrate the themes.

**Table 2.** Four main themes with corresponding sub-themes.

Theme	Sub-theme
(1) Expectations and outcomes of counselling	Coping strategies
	Acceptance
(2) Emotional impact of life with dementia	Being a burden to others
	Burden of coping
	Burden of other life events
(3) Appraisals of identity	Self or caring identity
	Self-care or resilience
	Importance of support network
(4) Importance of therapeutic relationship	Comfortable to disclose information
	Impartial listener
	Understanding of dementia

### Expectations and outcomes of counselling

#### Coping strategies

Participants caring for a relative with dementia felt a strong sense of urgency in seeking counselling and held certain expectations about enhancing their ability to cope. Value was placed on the intervention, and caregivers frequently discussed expectations around developing coping mechanisms or ‘*some ways of getting away from this*’, including both emotional and practical support.

*I’m left with the burden and everything and I don’t think it’s fair. And that’s what I want to learn to cope with now. You know, it makes me want to cry, I’m nearly crying now because it’s so upsetting.* (Caregiver: 10034).

Caregivers felt better equipped to cope with the caregiving role post-counselling. Counselling supported their ability to cope with the consequences of their relative’s diagnosis in various ways, including decision-making, shifting their perspective of caregiving and strategies to cope with everyday activities of the caregiving role. The therapist was seen as a source of support, who had encouraged participants to reflect on their lives in a different or new way.

*She’s got me to think about certain actions that I do, what the outcome would be, and sort of assess what would be the best strategy at this moment in time, and so I’ve done things different to what I would have done.* (Caregiver: 10003).

#### Acceptance

Most participants with dementia accepted the difficulties their diagnosis brought to their lives, including insight into expectations for their future. They were aware of limitations associated with their cognitive impairment, speaking in-depth about their anticipated decline.

*I’m having problems if I go out, I lose my bearings really. And I know the place well, but I just seem to have lost my bearings, I forget where I live and this that and the other* (PwD: 10046).

*You know it’s not bad really surviving and, more or less being able to, conversation. Provided that people are there who know you and can supply the word you want* (PwD: 10053).

One participant with dementia often compared their own situation with that of others, and this appeared to facilitate acceptance and the necessity of being realistic. Having insight into the progressive nature of dementia was a concern for participants.

*She could talk, but it was all very limited, and she had really – I mean she was obviously bad enough to have somebody with her […] she was just, seemed quite happy just sitting there. I thought gosh, I don’t really want to finish up like that. So, yeah, but you know, I think you’ve got to be realistic about it* (PwD: 10053).

Despite their insight, participants with dementia highlighted that they hoped counselling would allow them to explore their feelings associated with their diagnosis and subsequently facilitate their acceptance.

*I think because of the knowledge that I am going to die. Because I’ve got Alzheimer’s and having a terminal illness is quite a tough thing to face up to* (PwD: 10008).

Facilitating acceptance was not restricted to participants with dementia as family caregivers also noted that they hoped counselling would help them to accept their relative’s diagnosis and the associated consequences. By doing so, they hoped that acceptance would ease the burden of their caregiving role.

*The situation is only going to worsen, and I find that I’m actually grieving. Sort of I feel as though I’m in a phase of grieving, which I don’t seem to be able to get through finding acceptance […] I would like to feel as though I can accept the situation and I can move forward* (Caregiver: 10036).

The journey of acceptance appeared to be important in the development of resilience for participants. Having the opportunity to express their feelings and assess their situation with the counsellor seemed to help build confidence in their ability to face the challenges that the caregiving role can bring, reporting changes in mood and coping.

*It’s been wonderful. You know, it’s just a matter of – well, it doesn’t matter now because even if he deteriorates I know what I’m dealing with […] I think he accepts that he’s got dementia […] So I can deal with it, which makes my life easier as well as his* (Caregiver: 10033).

Post-intervention, both groups of participants acknowledged that the sessions had helped them to accept the dementia diagnosis and had ‘*become aware of what’s likely to happen over the years*’. This appeared to have a positive impact both psychologically and practically, despite information sharing not being a core component of the intervention.

*I think the word I’d probably use is, that I’ve felt a sense of acceptance and been – felt very safe* (Caregiver: 10047).

### Emotional impact of life with dementia

#### Being a burden to others

Participants with dementia were aware of their difficulties associated with dementia and appeared anxious about how this may negatively impact their relatives or friends within a caregiving role. Participants frequently perceived themselves as a ‘burden’ to those around them, acknowledging how their illness made life difficult for those around them.

*I feel with the people who are supporting me, that it would be a good thing if I did everything I could to sort myself out. I mean that’s the least they can expect of me […] I don’t know if I want to sort of burden them with it* (PwD: 10008).

One participant with dementia acknowledged her high dependence and appeared regretful that she believed she was burdensome to her relatives.

*My daughter look after me, I know that. Sometimes I think she spoil her life look after-old woman. You know, but what can you do. I’m here, I can’t do nothing […] I tried the washing up but I can’t* (PwD: 10055).

After attending counselling sessions, one participant with dementia discussed how the routine with his spouse had altered. He spoke positively about helping with daily activities within the home and clearly valued the new role he had adopted.

*I haven’t been washing up, since I’ve been to [counselling] I’ve started washing up for her on an evening, so when tea’s finished she can get ready watching the television […] so I usually try and do the washing up and put them away ready for her* (PwD: 10035).

Family caregivers also discussed their sense of being a burden to others. They frequently highlighted that although they had an existing support network, they found it difficult to express their negative feelings associated with the caregiving role to others. Participants often noted that others *had ‘enough going on*’, and they did not want to *‘burden them further’*.

*If I’m feeling quite down and I want to say, do you know what happened, they don’t want to know the full details and I think – no I shouldn’t really be giving them the details, you know, it’s not up to them to carry what I’m carrying* (Caregiver: 10007).

Post-counselling, many caregivers noted the benefits of being able to express themselves freely to an impartial listener. They believed that opening up to the counsellor about their difficulties within the caregiving role had a positive impact and was more beneficial than discussing their concerns with friends or family.

*I don’t always think it’s the best thing to just have friends and acquaintances to be telling your whole sort of in-depth things that are going on in your life. So, I think counselling –, sort of a step away, not being friends and things might be a better option […] it’s a better option to speak with somebody like that than speak with people that perhaps don’t want to, don’t really know what to say or don’t really know what to do for the best* (Caregiver: 10003).

In addition, one family caregiver acknowledged that by attending counselling sessions, she was able to spend quality time with her support network.

*I’ve had [counsellor] to offload to, I haven’t felt the need to be as much offloading to my friends, so it means that I’ve been able to have a bit more quality time with my friends rather than sitting and moaning about my Mum all the time* (Caregiver: 10032).

#### Burden of coping

The responsibility of caregiving was frequently highlighted as a stressful experience. The caregivers acknowledged dementia-related changes that were out of their control, and this often led to negative feelings as a result and a desire to escape or *‘walk away from it all*’. The most commonly mentioned were anxiety, stress and depression.

*I have had a couple of meltdowns in the last twelve months, those meltdowns don’t consist of anything violent, just huge crying sessions, depression, and if it continues any longer and it starts to go downhill that bit more, I can see something serious happening to my health* (Caregiver: 10021).

Loss and grief were also experienced by caregivers of people with dementia, relating to the ambiguous loss of their relative whilst they are still alive, but often with personality or emotional changes. This appeared to be in response to the compounded serial losses of varying magnitude during the trajectory of the dementia and the rapid nature of these changes, which often required significant adjustments.

*I’m experiencing a lot of the same feelings as grief, loss […] there’s a lot of that, it’s a sort of living grief* (Caregiver: 10047).

Caregivers often experienced a sense of guilt, related to both the expectation of their responsibility and a negative subjective appraisal of their own caregiving performance. As a result of this guilt, caregivers often experienced poor emotional and physical health and subsequently, a greater sense of caregiver burden. Some caregivers hoped that attending counselling would alleviate their sense of guilt and offer reassurance within their caregiving role.

*What I’d hope for is probably to, to get some validation that I know I’ve not done anything wrong, but there’s a lot of guilt associated with it, with looking after somebody who’s so poorly, you know, have I done the right thing by him, have I spent enough time with him* (Caregiver: 10047).

It was apparent that attending counselling sessions had helped to alleviate the feelings of guilt, and participants had reassessed their own appraisal of their caregiving performance. Individuals reflected that they had acted to the best of their ability and tried to avoid ruminating on *‘what if*’ situations.

*I found it very useful learning how to go through what I was feeling at each stage and address it with different techniques you know, and how I shouldn’t feel this way and it’s all part of a natural stage in life and not to feel too guilty really, so I found it helped with that […] I found it useful yeah, that whole phase, it’s helped me a lot* (Caregiver: 10062).

In addition to this emotional stress, the caregiving role often had adverse physical impacts. For some, in the absence of a social support network, caregivers felt alone in their role and acknowledged they were exhausted and overworked.

*It’s like the straw that breaks the camels back. Your health’s suffering, you’re not well, you’re not sleeping, you’re not eating properly, you’ve got headaches, you’re not dealing with things properly […] I don’t have enough hours in a day, I don’t have enough time to do anything* (Caregiver: 10049).

As the dementia progressed, some caregivers noted negative changes in their own personality and behaviours. For example some reported becoming progressively frustrated, irritable and angry, which concerned them. A few caregivers reported that they hoped counselling sessions would help them to manage the negative changes they were experiencing themselves due to their caregiving role and respond to their relative in a more positive way.

*It’s me with my temper, I’m afraid. Defending myself actually. I’m not bad tempered all the time, don’t think like that because I’m not, but I get very, very frustrated and irritated* (Caregiver: 10064).

Lastly, the burden of coping was apparent for people living with dementia. All participants expressed negative feelings associated with their diagnosis, including lack of understanding from those around them. Many were seeking counselling to alleviate these emotions and be able to ‘move forward’ with their lives post-diagnosis.

*I just want to forget things now and start living again. I’m not living. I’m surviving* (PwD: 10046).

One participant with dementia clearly expressed how much he valued the counselling sessions. He spoke about how he felt more positive for the future and the negative feelings associated with his diagnosis had lessened.

*It’s made me think more, and think about myself because I’m not one of these ones – I worry about everyone else apart from myself […] and I sort of, I’ve locked myself away. I really did […] I knew I needed help and I’m glad I’ve got it. I’m more open now and looking for- I’m looking forward to sort of, the future now* (PwD: 10046).

#### Burden of other life events

Family caregivers frequently mentioned that counselling would be beneficial not only to discuss their caregiving role but also to express their feelings about their competing responsibilities. The experience of adverse life events outside of the caregiving role, such as interpersonal problems with relatives, interpersonal losses, health problems and work/financial issues, impacted on the participants’ ability to cope. For some, an event unrelated to their relative’s diagnosis, such as another death within the family, had become the ‘tipping point’ that led them to seek help.

*I’m a mum so I run a household, I also work part-time, and I also am a volunteer. And I’m also setting up a business so it’s really tough being a carer […] obviously emotionally hugely but from a practical side* (Caregiver: 10047).

Those that spoke frequently about other life events highlighted how advantageous counselling had been. They expressed that they had new coping strategies in place and were better equipped to deal with both their caregiving role and other life events, through improved emotional responses.

*They helped me to accept the situation that I’m in and not fight against it […] a bit more open-minded about it now, a few different ways of looking at it, where before I was just angry* (Caregiver: 10034).

### Appraisals of identity

#### Self or caring identity

The role of caregiver and corresponding responsibilities had started to consume the participants, leaving limited time available for other activities and behaviours that may have defined the person prior to adopting the caregiver role. Through the responsibilities of the caregiving role, caregivers often relinquished other roles deemed not as urgent or important, which appeared to impact their sense of personal identity. For many participants, the caregiving role had become their dominant identity, perceiving that they had *‘given everything up’.*

*And this has hit me in that sense that I’m now looked at as his carer […] and it all sounds a bit, I don’t know if it sounds strange to you* (Caregiver: 10007).

Furthermore, caregivers reported a desire to maintain the relationship they had shared with their relative before adopting the caregiving role, demonstrating a loss of shared identity. For several caregivers, their role had changed from daughter or son to an almost parental role, and this shift in roles and the relationship was difficult to process.

*I don’t get treat like a daughter, it’s almost like that role has gone – I’m just like her carer, mum figure now for her. You know, which is a bit sad* (Caregiver: 10032).

If the relative with dementia moved into a care setting or passed away, participants struggled readjusting to an identity without the primary caregiving role. This was particularly apparent where the loss was sudden, and individuals had not had the time or space to reflect on this, with caregiving described as *‘a way of life’* that was suddenly removed in a *‘jolt’.*

*You don’t realise you’re freed up of caring until a few weeks has passed. You kind of wake up and think oh, I must go round, and then you forget oh, she’s not there – she’s somewhere else* (Caregiver: 10062).

Given the struggles around caregiving identity, counselling appeared to allow caregivers to explore their feelings towards their relative, whilst facilitating them to reconnect with other aspects of the self and provided them with permission to refocus their attention.

*Yeah, look after myself. I said to [counsellor] that I need to take the advice on board. And that’s what I said today. I have taken advice on board, I have put things in place so that I can get away if I want for a while. And I think that’s a good thing* (Caregiver: 10005).

#### Self-care or resilience

Participants talked about a lack of focus on, or time for, themselves due to the caregiving role. Many caregiver participants suggested that counselling enabled them to think about and reflect on their own feelings and needs. For some, counselling helped them see that they had negative feelings towards themselves.

*We talked about it at the last session and it’s that I’ve got to concentrate on myself because I’ve kind of let myself go, so I’ve got to get myself sorted out with you know eating and doing a routine really. That’s what I’ve got to do now* (Caregiver: 10059).

Some participants thought that an increased focus on the self would occur during counselling sessions as they had dedicated *‘time for me’.* This highlighted the lack of time they were able to spend on their own generally.

*I want to have that really fabulous luxury of actually having some time and space for me just to talk to somebody who knows nothing about us […] and that just feels really like a blessing* (Caregiver: 10047).

Balancing the needs of the person with dementia and their own needs was challenging, with counselling allowing participants the opportunity to reflect on this balance and identify where this had become problematic or maladaptive.

*How much do I consider my dad and how much do I consider my own mental health?* (Caregiver: 10047).

#### Importance of support network

Support from networks including friends, relatives and professionals appeared to be integral to the participants’ caring role. Each relationship seemed to have a certain purpose and provided different sources of support ranging from practical to emotional. For example participants gained valuable practical support from relatives as they were experiencing the same situation. Where this support was absent, many caregivers voiced their difficulties adopting the caregiving role alone. Sibling relationships were frequently mentioned, either as a supportive network who as a team provided support and had *‘unsaid “it’s your turn”’* arrangements, or due to issues around inequality in caregiving responsibilities and workload.

*I just don’t understand how my brother, when he’s had a good mother that’s always cared for him, looked after him […] not to care how she is, when it’s cold, you know just leave it to someone else. I can’t get my head round why anyone can be so, so cold. And so selfish and everything that I hate, I just hate him* (Caregiver: 10034).

Overall, participants emphasised the need for comprehensive support provided by a range of people. For those who did not have a strong social support network, it was apparent they had both emotional and practical difficulties that they could not share with anyone. Thus, the therapeutic relationship is essential as a support system that may fill the void that some participants may feel, resulting from an absent support network.

### Importance of therapeutic relationship

#### Comfortable to disclose information

An essential element of counselling was the connection established between each participant and the counsellor. Once this connection was made during sessions, participants appeared to be comfortable and willing to disclose information and accept help. For most participants, the connection with the counsellor was easy to establish within the early sessions, and they felt comfortable to disclose information straight away.

*That comes out from [counsellor], she’s very good at bringing it out and she’s not pushing you at all. Very open and positive* (PwD: 10053).

However, establishing the therapeutic relationship was challenging for some as a few participants expressed their reluctance to disclose information to a ‘stranger’. Furthermore, some participants did not understand exactly how counselling could help them or did not know whether they would be able to *‘open up’* to the counsellor. This was particularly the case for people with dementia whose caregiver had recently completed counselling and then referred them to the service.

*I’m quite a private person really. So, it’s a little bit difficult as I say […] I’d just go and see whether it helps in any way. This is what I think, I don’t think anybody can help* (Caregiver: 10005).

Despite this, the participants who expressed some reluctance prior to counselling highlighted how they were comfortable to disclose information once they had met the counsellor and gradually established a good connection.

*Well it was difficult because I don’t normally talk to anybody much. You know, not in person and stuff like that. But I found that the person was really, you know, on the ball of making people feel comfortable, you know* (PwD: 10035).

#### Impartial listener

Participants identified the benefits of talking to an impartial listener, with value placed on being able to talk openly and freely to someone outside of their existing support network. This was of particular importance as participants were reluctant to share their feelings with their relatives and friends to avoid burdening them. In addition, many participants understood that their support network were either too emotionally attached to the situation, offered their own personal experiences instead of listening to the current situation or did not have sufficient knowledge of the impact of dementia and lacked understanding.

*With* [counsellor] *being trained to do this, you know, it’s a better option to speak with somebody like that then speak with people that perhaps don’t want to – don’t really know what to say or don’t really know what to do for the best* (Caregiver: 10003).

Being able to talk to someone neutral and non-judgemental appeared to help normalise the participants’ feelings and emotions, and it was an essential element of the counselling experience for both groups of participants.

*She explained what was going to happen, it all sounded quite right. She above all, I think what, I noticed most with her – here is somebody who is actually going to listen to what I say, and take some notice* (PwD: 10053).

*It’s just good to have someone to talk to, and that understands and listens and doesn’t judge* (Caregiver: 10004).

#### Understanding of dementia

All participants were helped by the counsellor’s accurate understanding of dementia and the impact of living with dementia. It appeared that having access to a knowledgeable listener provided relief to both caregivers and people with dementia and was an essential part of the sessions. One caregiver also expressed that it was particularly beneficial as they were able to begin discussing their emotions and concerns straight away, rather than having to first explain the impact of living with, or caring for somebody with dementia. Participants reflected on this as a dichotomy from sharing experiences with friends and family, where they frequently were required to explain the diagnosis and symptoms in detail.

*I think it is good to have an experience or understanding of dementia because it’s so complicated and there’s so many things – dynamics to it. I don’t want to sit in a session and talk about, you know, general dementia because it feels like I’m wasting – you know time is precious at counselling, so I feel like I want to get to the nitty gritty quite quickly* (Caregiver: 10048).

Therapist understanding of dementia was perceived as important as they were able to offer different perspectives regarding the caregiving role and the challenges that come with a dementia diagnosis. Participants who initially felt a range of negative emotions could see how the counselling process had helped to improve their situation. Being able to separate behaviours associated with dementia and the person themselves was beneficial for participants.

*I found it useful to think about my dad’s behaviours because of his dementia. And that he couldn’t help it. That was very helpful for me because I was so frustrated by my dad’s behaviours. And we talked a lot about, you know- are you annoyed with your dad or are you annoyed with the dementia, you know, so we kind of separated the two out which helped a lot* (Caregiver: 10048).

Participants expressed that the sessions had been more beneficial as the counsellor was knowledgeable of dementia, and the sessions may not have been as helpful if this understanding was absent.

*To explain what may happen when somebody has dementia and how dementia progresses as well she just knew. You know, she understood, and I was so – it was good that she had that experience really good, whereas a general counsellor may not have had that* (Caregiver: 10048).

## Discussion

The present study explored participant experiences and expectations of counselling for those affected by dementia. There are clearly many elements within the counselling process that address various needs for both people living with dementia and their caregivers. This article provides an insight into how counselling can benefit this client population and builds a foundation to inform both counsellor practice and the development of future research within the field of counselling and dementia.

The findings in this study highlight the strong emotional impact of the diagnosis of dementia for both participant groups, particularly in response to the unknown trajectory of the dementia, the grief associated with actual and anticipated losses and a sense of helplessness or loss of self as someone living with dementia or caring for a relative with dementia. These current findings are consistent with previous literature that explores the perceptions of the emotional impact of a dementia diagnosis (Aminzadeh et al., 2007) and with the three key areas that people with dementia have highlighted as recommended focus for therapeutic interventions; loss of abilities/identity, coping mechanisms and support (Birtwell & Dubrow-Marshall, 2018). It appeared that change in the identity of people with dementia, as perceived by the caregivers, was associated with caregivers’ reported change in sense of their own identity and their perception of the dyadic relationship, in line with previous literature that the onset and progression of dementia is likely to result in a change in the interpersonal relationship (Hayes et al., 2009; Noyes et al., 2009).

Thus, it is important for psychotherapeutic strategies to be implemented to create a more supportive context for people with dementia and their caregivers (Aminzadeh et al., 2007). Given the threat of loss of identity, counselling can help to highlight the individual’s capabilities and strengths and provide opportunities for personal development and enhancing a sense of personhood (Bryden, 2002; Cheston et al., 2003). Sabat and Harré (1992) argued that identity can be created through talk and social interaction, subsequently reducing the social isolation of the person with dementia (Jones, 1995). Furthermore, such interventions can support an individual’s sense of self, increase self-acceptance and can have positive effects on depression, anxiety and caregiver burden (Birtwell & Dubrow-Marshall, 2018; Elvish et al., 2014; Mittelman et al., 2007).

Many people with dementia have traditionally been regarded as ‘beyond therapeutic reach’ (Sommerbeck, 2006) and thus unable to establish a therapeutic relationship. Moreover, it is well documented that older adults underutilise professional psychological services (Robb et al., 2003). Brownlie (2009) noted that age was a key variable when exploring attitudes towards counselling, and older adults were less open to the possibilities of therapeutic intervention than those in younger age groups. Subsequently, counselling or psychotherapeutic interventions may be underutilised for people with dementia. However, research has explored psychotherapeutic interventions for people with dementia and indicated positive impacts (e.g. Perren & Richardson, 2018). Additionally, whilst clinicians are aware that many people with dementia, especially those with mild cognitive impairment, are able to communicate effectively, no therapeutic interventions have been tested with those with more severe dementia who would be expected to have increased cognitive impairment (Shoesmith et al., 2020).

In the current study, both participant groups reported positive change post-counselling. Caregivers frequently reappraised their performance within the caregiving role, had an alleviated sense of grief and reported enhanced coping strategies. Likewise, participants with dementia often valued the counselling process in terms of acceptance and being able to ‘move forward’. However, modifications are required to ensure that people with dementia can effectively engage with counselling, such as simplifying any materials, using techniques that help participants retrieve information from previous sessions and involvement of caregivers where appropriate (Tay et al., 2019).

It was clear in this study that both participant groups could establish a therapeutic relationship with the counsellor, and this was an essential element for all participants. This is in line with thoughts that the relationship between a therapist and client is more important than the mode of delivery or modality (Paul & Charura, 2014). Factors such as empathy, warmth and acceptance are thought to be key for counselling professionals (Orlinsky et al. 2004; Norcross and Lambert, 2018). Consistent with previous literature, understanding, trust and non-judgement from the counsellor was vital (Elvish et al., 2014). However, Cooper (2010) argues that whilst a good therapeutic relationship is predictive of good therapeutic outcomes, this is not something that the counsellor ‘provides’, but rather something that emerges in the interaction between the client and counsellor. This interaction may have been facilitated by the counsellor’s knowledge and awareness of dementia as this was reported by participants as particularly valuable and appeared to be an essential element for successful engagement in counselling.

Specific dementia training should be sought by practitioners wishing to engage with those living with, and supporting those with, dementia.

In the present study, participants reported valuing the counselling intervention. However, there is still limited evidence for the effectiveness of such interventions for people with dementia. Future research should test the effectiveness and cost-effectiveness of counselling interventions, using robust methods. Practical considerations such as optimal session and intervention length, preferred modality, attrition and caregiver involvement should be explored (Shoesmith et al., 2020; Tay et al., 2019). Process evaluations should be incorporated, allowing understanding of how interventions lead or do not lead to changes for participants and the mechanisms through which this change operates (Moore et al., 2015). Recommendations for practice are presented in our recent systematic review and include the development of generic, core and specialist competencies and guidance for practitioners on working with those with cognitive impairment (Shoesmith et al., 2020).

## Limitations

There are several limitations associated with the present work. Firstly, counselling was delivered by a single individual, and therefore, these findings may not be generalisable to counselling services more widely. Secondly, this study only considers the perspectives of the participants attending counselling sessions as opposed to the perspectives of the counsellor. The perceptions of those attending and those delivering may not always align. However, a full discussion of these issues is presented in [blinded]. Thirdly, most of the participants identified as white British and were recruited through a single faith-based organisation, and therefore, the findings are not generalisable more widely. However, the present research demonstrates that faith-based organisations are a feasible location for counselling interventions for people living with dementia and their families. This has implications for access to services for groups who are sometimes perceived to be ‘hard to reach’, by working with organisations who regularly provide support to these individuals. Lastly, we recruited fewer participants with dementia than caregivers. Despite this, the richness of the data provides an insight into the perspectives of people with dementia participating in counselling sessions and builds a foundation for the development of practice and research in this area.

## Conclusions

In conclusion, the present study demonstrated that the relational counselling intervention was able to address a range of needs and concerns for people with dementia and caregivers. However, concerns have been raised about whether people with dementia are able to fully engage with such interventions and further research is needed to explore this in depth, across counselling modalities, to establish the appropriateness and effectiveness of counselling. Despite this, at a time where no cure is available for dementia, psychotherapeutic interventions are able to offer ongoing support for those living with, and supporting those with, the condition.
